# Quantification of [^11^C]PBR28 data after systemic lipopolysaccharide challenge

**DOI:** 10.1186/s13550-020-0605-7

**Published:** 2020-03-12

**Authors:** Eric A. Woodcock, Martin Schain, Kelly P. Cosgrove, Ansel T. Hillmer

**Affiliations:** 1 Department of Pscyhiatry, Yale School of Medicine, 300 George St., New Haven, CT USA; 2 Neurobiology Research Unit, Copenhagen University Hospital, Copenhagen, Denmark; 3 Department of Radiology & Biomedical Imaging, Yale School of Medicine, 330 Cedar St., New Haven, CT USA

**Keywords:** [^11^C]PBR28 PET imaging, TSPO, Neuroimmune, Neuroinflammation, Lipopolysaccharide

## Abstract

**Background:**

Lipopolysaccharide (LPS) is a classic immune stimulus. LPS combined with positron emission tomography (PET) 18 kDa translocator protein (TSPO) brain imaging provides a robust human laboratory model to study neuroimmune signaling. To evaluate optimal analysis of these data, this work compared the sensitivity of six quantification approaches.

**Methods:**

[^11^C]PBR28 data from healthy volunteers (*N* = 8) were collected before and 3 h after LPS challenge (1.0 ng/kg IV). Quantification approaches included total volume of distribution estimated with two tissue, and two tissue plus irreversible uptake in whole blood, compartment models (2TCM and 2TCM-1k, respectively) and multilinear analysis-1 (MA-1); binding potential estimated with simultaneous estimation (SIME); standardized uptake values (SUV); and SUV ratio (SUVR).

**Results:**

The 2TCM, 2TCM-1k, MA-1, and SIME approaches each yielded substantive effect sizes for LPS effects (partial *η*^2^ = 0.56–0.89, *p*s <. 05), whereas SUV and SUVR did not.

**Conclusion:**

These findings highlight the importance of incorporating AIF measurements to quantify LPS-TSPO studies.

## Introduction

Positron emission tomography (PET) imaging of the 18 kDa translocator protein (TSPO) provides a quantitative measure of an in vivo neuroimmune system marker [1]. While interpretation of baseline TSPO levels is complicated by several factors [2], TSPO response after lipopolysaccharide (LPS) challenge yields a measurement of “neuroimmune response” to an acute immunogenic stimulus. LPS is gram-negative bacteria that evokes classic pro-inflammatory responses via the toll-like receptor-4 complex (TLR4). Preclinical PET studies indicate intra-striatal LPS injection increases TSPO levels relative to contralateral striatal levels and saline-injected controls; findings confirmed by autoradiography and cold-tracer studies [3, 4]. Prior research demonstrates LPS increases brain TSPO levels across species, including rodents [3, 4], nonhuman primates [5], and humans [6]. Thus, LPS challenge provides a robust experimental model for investigating neuroimmune signaling in people.

The dramatic LPS effects on specific binding motivate reanalysis and confirmation of quantification approaches. In most cases, reference region approaches are not appropriate for full quantification of TSPO radioligands due to the lack of regions devoid of TSPO in the brain, although pseudo-reference region approaches have been validated for specific scenarios [7]. In this study, we evaluated the sensitivity of different TSPO quantification approaches to LPS effects, with careful consideration of approaches incorporating an arterial input function, using previously reported data with the second-generation PET TSPO radiotracer [^11^C]PBR28 [6]. Specifically, we evaluated analytic approaches which incorporate an arterial input function (AIF): total volume of distribution (*V*_T_) estimated with a two-tissue compartment model (2TCM), a 2TCM variant which includes a parameter purported to describe irreversible uptake in endothelial cells (2TCM-1k) [8], multilinear analysis-1 (MA-1) [9], and estimation of binding potential (BP_P_) with simultaneous estimation (SIME) [10]. We also evaluated semi-quantitative metrics that do not incorporate an AIF: standardized uptake values (SUV) and SUV ratio (SUVR). We hypothesized that models that incorporate the AIF (2TCM, 2TCM-1k, MA-1, and SIME) would be more sensitive to LPS-induced TSPO increases.

## Methods

### Recruitment

The Yale University School of Medicine Human Investigation Committee and the Radioactive Drug Research Committee approved all study procedures. Subjects were genotyped for the rs6971 polymorphism: only “high-” and “mixed-affinity binders” were eligible (HABs and MABs, respectively). Subjects (*N =* 8; 5 MABs, 24.9 ± 5.5 years old, 87.5 ± 12.3 kg, 8 M) were recruited, screened, and enrolled as previously described [6]. All subjects provided written informed consent.

### Experimental procedures

All subjects participated in one experimental session consisting of two 120-minute [^11^C]PBR28 PET scans on the same day. Following the baseline [^11^C]PBR28 PET scan, subjects were injected with LPS (1.0 ng/kg IV), NIH Clinical Center Reference Endotoxin *E. coli* serotype O:113. The second [^11^C]PBR28 PET scan started 3 h after the LPS injection.

### Data processing

PET acquisition details have been fully described elsewhere [11]. Briefly, [^11^C]PBR28 was synthesized with high molar activity 569 ± 327 MBq/nmol (15.4 ± 8.8 mCi/nmol). [^11^C]PBR28 was injected via slow bolus (1 min), and PET data were acquired for 120 min on the high resolution research tomograph (HRRT, Siemens) with simultaneous optical head motion tracking (Vicra, NDI Systems). Dynamic list-mode data were histogrammed into intervals ranging from 30 s to 5 min and reconstructed using the MOLAR algorithm. T_1_-weighted structural MR images were coregistered to PET data for region of interest (ROI)-based extraction of time-activity curves (TACs) determined in AAL template space. ROIs assessed included the caudate, cerebellum, hippocampus, thalamus, putamen, and frontal, parietal, temporal, and occipital cortices. Arterial blood samples were collected throughout each 120-minute scan to measure the metabolite-corrected AIF and plasma free fraction (ƒ_p_), as previously described [6, 12].

### Analytic approaches

Area under the curve (AUC) of the metabolite-corrected AIF was calculated using numerical trapezoidal integration. Imaging data were analyzed using each approach: 1TCM, 2TCM, 2TCM-1k, MA-1, SIME, SUV, and SUVR. Plasma uptake delay (τ) was estimated using a 1TCM from the first 10 min of data and was fixed for 2TCM and 2TCM-1k analyses. Compartment modeling analyses were performed with the Compartment Model Kinetic Analysis Tool (COMKAT [13]) in the MATLAB environment. 1TCM poorly described ROI TACs; therefore, results are not reported. In the 2TCM model, four rate constants were estimated: *K*_1_, *k*_2_, *k*_3_, and *k*_4_ [14]. The 2TCM-1k model includes a fifth parameter (*k*_b_) that models purported irreversible uptake in endothelial cells [8]. The corrected Akaike Information Criterion (AICc [15]) indicated model preference for a fixed blood volume fraction (*V*_b_ = 5%) for 2TCM and 2TCM-1k. For MA-1 [9], *V*_T_ was estimated using t* = 30, consistent with prior work [11]. Simultaneous estimation (SIME) simultaneously fits TACs across all ROIs to estimate whole-brain *V*_ND_ which, in combination with regional *V*_T_ values, can estimate ROI binding potentials specific to total plasma concentration (BP_P_) [10]. Due to the low free fraction (~ 2%) resulting in poor *f*_P_ precision [16], analyses incorporating ƒ_p_ in *V*_T_ estimates are only included for completeness as Additional file 1. Finally, SUV was calculated as mean tissue activity concentration for each ROI during specified timeframes (60–90 min, 90–120 min) normalized by subject body weight and injected [^11^C]PBR28 dose. SUVR was estimated by dividing ROI SUV by whole-brain SUV. AICc was used to compare model parsimony for 2TCM vs. 2TCM-1k [15].

Repeated measures analyses of variance (rmANOVA) were used to evaluate LPS effects on each calculated endpoint (statistical transformations applied as needed to normalize distributions) across ROIs (within-subject factor) with rs6971 genotype (HAB vs. MAB) as a between-subject factor (significance threshold: *p* < .05). Partial eta-squared (*η*^*2*^) effect sizes were estimated from rmANOVAs.

## Results

**Fig. 1:**
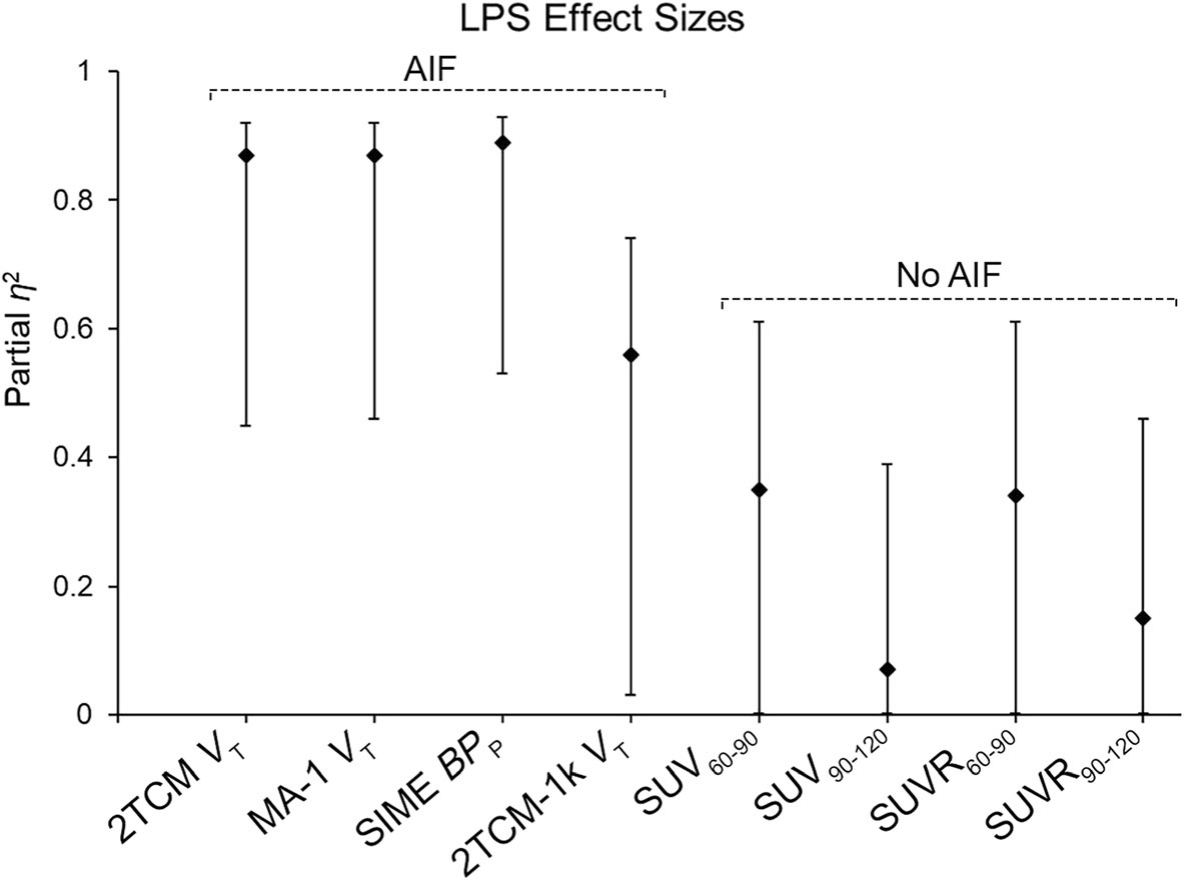
LPS effect sizes (partial *η*^2^) and 95% confidence intervals for each analytic approach are depicted

**Fig. 2:**
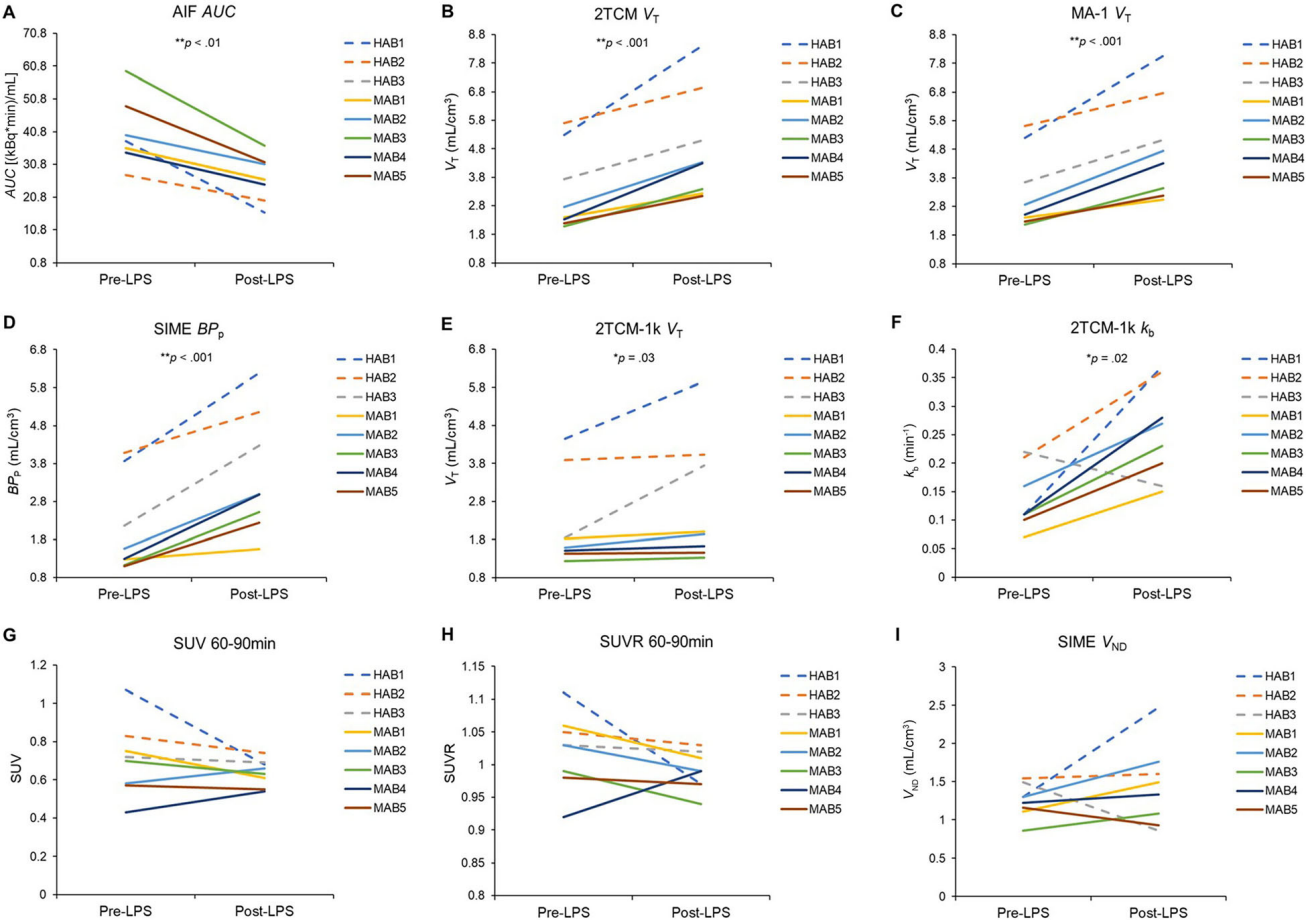
Individual subject data are depicted as means across ROIs (HABs = dashed lines; MABs = solid lines). **a** AIF AUC. **b** 2TCM *V*_T_. **c** MA-1 *V*_T_. **d** SIME BP_P_. **e** 2TCM-1k *V*_T_. **f** 2TCM-1k *k*_*b*_. **g** SUV 60–90 min. **h** SUVR 60–90 min. **i** SIME *V*_ND_

The AIF AUC significantly decreased after LPS across rs6971 genotypes (*F* (1, 6) = 41.06, partial *η*^*2*^ = 0.87; Fig. 2a; group average time curves shown in Additional file 1: Figure S1). In the brain, 2TCM *V*_T_ and 2TCM-1k *V*_T_ (inverse-transformed) significantly increased after LPS by 47% and 24% on average, respectively (2TCM: *F* (1, 6) = 38.39, partial *η*^*2*^ = 0.87; 2TCM-1k: *F* (1, 6) = 7.55, partial *η*^*2*^ = 0.56; Table 1; Figs. 1 and 2). Mean AICc values indicated 2TCM was preferred to 2TCM-1k (2TCM, 20.0 ± 0.49 and 20.3 ± 0.78; 2TCM-1k, 23.5 ± 0.75 and 23.89 ± 1.38; pre- and post-LPS, respectively). MA-1 *V*_T_ and SIME BP_P_ significantly increased after LPS by 45% and 82% on average, respectively (*F* (1, 6) = 39.02, partial *η*^*2*^ = 0.87, and *F* (1, 6) = 49.29, partial *η*^*2*^ = 0.89, respectively). Importantly, whole-brain SIME *V*_ND_ did not significantly change from pre- to post-LPS (*p* = .39). SIME BP_P_, 2TCM *V*_T_, and MA-1 *V*_T_ exhibited the largest LPS effects (partial *η*^*2*^ = 0.87–0.89; Table 1; Fig. 1; Additional file 1: Figure S2). SUV and SUVR estimated smaller LPS effects (partial *η*^*2*^ ≤ 0.35), which indicated an apparent decrease in TSPO levels from baseline.

**Table 1:** LPS effects

Model	Parameter	LPS Effect				
		Partial *η*^*2*^	95% CI	Overall (%)	MABs (%)	HABs (%)
AIF AUC	AUC	0.87**	0.47–0.92	− 32.6	− 30.0	− 38.1
2TCM	*V*_T_	0.87**	0.45–0.92	46.7	56.4	38.9
2TCM-1k	*V*_T_	0.56*	0.03–0.74	24.3	10.4	34.6
MA-1	*V*_T_	0.87**	0.46–0.92	44.9	53.3	37.8
SIME	BP_P_	0.89**	0.53–0.93	81.9	94.1	61.5
SUV, 60–90 min	SUV	0.35	0.00–0.61	− 9.9	− 1.4	− 19.8
SUV, 90–120 min	SUV	0.07	0.00–0.39	− 1.5	8.7	− 13.6
SUVR, 60–90 min	SUVR	0.34	0.00–0.61	− 3.0	− 1.4	− 5.5
SUVR, 90–120 min	SUVR	0.15	0.00–0.46	− 1.3	− 0.7	− 4.3

LPS effect % = [(Post-LPS − Pre-LPS)/Pre-LPS] × 100. Partial *η*^*2*^ effect size interpretation: “Small” ≤ 0.09, “Moderate” = 0.10–0.24, “Large” ≥ 0.25. *95% CI* 95% confidence interval for the partial *η*^2^ effect size at *p* = .05. Significant LPS effects are noted

*p ≤ .05

**p < .01

## Discussion

This work indicates that peripheral LPS administration significantly reduced the [^11^C]PBR28 AIF AUC by 33%. Approaches that incorporate the AIF (2TCM *V*_T_, 2TCM-1k *V*_T_, MA-1 *V*_T_, and SIME BP_P_) yielded large effect sizes for TSPO increases following LPS administration. The SIME, 2TCM, and MA-1 approaches yielded nearly identical sensitivity (partial *η*^*2*^ = 0.87–0.89), while 2TCM-1k was less sensitive, albeit still yielding a large effect size (partial *η*^*2*^ = 0.56). AICc values preferred 2TCM to 2TCM-1k. SIME estimates of BP_P_ are directly proportional to TSPO levels (i.e., do not include nondisplaceable uptake) and therefore provide theoretically improved estimates of specific binding. BP_P_ yielded the largest percentage increase following LPS challenge (82%), but also greater variability and thus a nearly identical effect size as 2TCM *V*_T_ and MA-1 *V*_T_. Notably, *V*_ND_ estimated with SIME did not change pre- to post-LPS. Estimation of *V*_T_/ƒ_p_ also yielded significant LPS-induced increases (Additional file 1) but smaller effect sizes than *V*_T_ due to the variability in ƒ_p_ estimation. No evidence for LPS effects on ƒ_p_ measurements was found. Therefore, we conclude that 2TCM, MA-1, and SIME are the most sensitive quantitative approaches to estimate TSPO availability in the context of this LPS paradigm.

In contrast, semi-quantitative approaches that do not incorporate AIF measurements (SUV and SUVR) failed to detect significant LPS-induced TSPO increases in the brain. The significant reduction in AIF AUC suggests increased [^11^C]PBR28 specific binding in the periphery and may explain the poor performance of SUV in this context. The global [^11^C]PBR28 *V*_T_ increase after LPS confirms the lack of suitable reference region for TSPO in this context and contributed to the poor performance of SUVR. Taken together, these findings highlight the importance of the metabolite-corrected AIF for LPS challenge studies, and support previous cautionary conclusions in the use of SUV-based quantification of TSPO [17, 18].

LPS is a classic immune stimulus shown to evoke robust neuroimmune responses. Preclinical findings indicate LPS increased brain TSPO levels which were co-localized with activated microglia (CD11b and OX2 immunoreactivity) and astrocytes (GFAP immunoreactivity), increased expression of toll-like receptors (TLR-2 and TLR-4), and increased brain cytokine levels [3, 4, 19]. LPS administration substantially increases TSPO immunohistochemical markers, mRNA levels, and protein expression in rodents [20, 21]. PET imaging studies confirm that LPS upregulates TSPO levels across species [3–5], including humans [6]. In sum, this literature strongly supports our expectation that brain TSPO should increase in response to systemic LPS administration.

Limitations of this work include our inability to confirm whether LPS activates microglia and/or astrocytes and recruits additional TSPO-expressing cells, or any combination of these or other properties [22]. Additionally, future research is needed to investigate if less invasive approaches, i.e., venous input functions, can replace the AIF.

In conclusion, our findings indicate that analytic approaches that incorporate the AIF are necessary to detect LPS effects on brain TSPO levels. The findings highlight the importance of the metabolite-corrected AIF for quantification of LPS-induced [^11^C]PBR28 brain changes.

## Supplementary information

**Additional file 1:** Supplemental Material. **Table S1.Figure S1.** Mean AIF data are depicted separately for rs6971 genotype HABs (C/C; gray lines) and MABs (C/T; black lines) pre-LPS (solid lines) and post-LPS (dashed lines). **Figure S2.** Individual values, pre- and post-LPS, are depicted for each brain region for models that incorporate the AIF: A) 2TCM VT; B) 2TCM-1k VT; C) MA-1 VT (t*=30); and D) SIME BPP. The same color marker was used to depict each subject’s data across models and LPS dose (pre- vs. post-LPS). **Figure S3.** A Time-Activity Curve was extracted from the occipital cortex (OCC) of a representative subject and kinetic model fit are depicted: A) pre-LPS 2TCM; B) post-LPS 2TCM; C) pre-LPS 2TCM-1k; and D) post-LPS 2TCM-1k.

## Data Availability

Due to the sensitive nature of human participant information, data are available upon reasonable request by contacting the corresponding author.

## Abbreviations

2TCM: Two-tissue compartment model
AICc: Akaike information criterion corrected
AIF: Arterial input function
AUC: Area under the curve
BP: Binding potential
ƒ_p_: Plasma free fraction
HRRT: High resolution research tomograph
LPS: Lipopolysaccharide
MA-1: Multilinear analysis-1
PET: Positron emission tomography
ROI: Region of interest
SIME: Simultaneous estimation
SUV: Standardized uptake value
SUVR: Standardize uptake value ratio
TACs: Time-activity curves
TLR2: Toll-like receptor-2
TLR4: Toll-like receptor-4 complex
TSPO: 18 kDa translocator protein
*V*_T_: Total volume of distribution
