# Supplementary material for: Quantification of [11C]PBR28 data after systemic lipopolysaccharide challenge
Source: EJNMMI Res. 2020 Mar 12;10:19. doi: 10.1186/s13550-020-0605-7 (PMC7067964; doi:10.1186/s13550-020-0605-7)
Supplement: Supplementary file 1 — Additional file 1: Supplemental Material. Table S1.Figure S1. Mean AIF data are depicted separately for rs6971 genotype HABs (C/C; gray lines) and MABs (C/T; black lines) pre-LPS (solid lines) and post-LPS (dashed lines). Figure S2. Individual values, pre- and post-LPS, are depicted for each brain region for models that incorporate the AIF: A) 2TCM VT; B) 2TCM-1k VT; C) MA-1 VT (t*=30); and D) SIME BPP. The same color marker was used to depict each subject’s data across models and LPS dose (pre- vs. post-LPS). Figure S3. A Time-Activity Curve was extracted from the occipital cortex (OCC) of a representative subject and kinetic model fit are depicted: A) pre-LPS 2TCM; B) post-LPS 2TCM; C) pre-LPS 2TCM-1k; and D) post-LPS 2TCM-1k. [file 13550_2020_605_MOESM1_ESM.zip › 13550_2020_605_MOESM1_ESM/Supplemental_Material_2.13.20_nofigs.docx]

**Supplemental Material**

Supplemental analyses evaluated the ratio of *V*_T_ to plasma-free fraction (*V*_T_ / ƒ_p_) for 2TCM, 2TCM-1k, and MA-1 models. Repeated measures analyses of variance (rmANOVA) were used to evaluate LPS effects across ROIs (within-subject factor) with rs6971 genotype (HAB vs. MAB) as a between-subjects factor (significance threshold: *p*≤.05). Also, rmANOVA evaluated LPS effects on plasma-free fraction with rs6971 genotype as a between-subject factor. Finally, rmANOVA evaluated LPS effects on *V*_T_ for 2TCM and 2TCM-1k models, in which *V*_b_ was estimated rather than fixed (*V*_b_ = 5%) as in the main text.

LPS significantly increased *V*_T_ / ƒ_p_ as estimated via 2TCM, 2TCM-1k, and MA-1 models (2TCM: *F*(1,6)=9.18, partial *η^2^* = 0.61; 2TCM-1k: *F*(1,6)=13.51, partial *η^2^* = 0.69; MA-1: *F*(1,6)=9.31, partial *η^2^* = 0.61, respectively; Supplemental Table 1). rmANOVA indicated LPS did not alter ƒ_p_ (*p*=.92; pre-LPS = 3.06 ± 0.65%; post-LPS = 2.99 ± 0.86%). LPS significantly increased *V*_T_ as estimated via 2TCM and 2TCM-1k when *V*_b_ was estimated (2TCM: *F*(1,6) = 41.56, partial *η^2^* = 0.87; 2TCM-1k: *F*(1,6) = 8.46, partial *η^2^* = 0.59).

Supplemental Table 1

|  |  | **Pre-LPS** | | **Post-LPS** | | **LPS Effect** | | | | |
| --- | --- | --- | --- | --- | --- | --- | --- | --- | --- | --- |
|  | **Parameter** | **MABs** | **HABs** | **MABs** | **HABs** | **Partial *η^2^*** | **95% C.I.** | **Overall %** | **MABs %** | **HABs %** |
| 2TCM | *V*_T_ / ƒ_p_ | 84.4 (11.0) | 141.1 (19.5) | 139.9 (21.5) | 207.5 (27.2) | 0.61* | 0.07-0.77 | 58.7% | 65.7% | 47.1% |
| 2TCM-1k | *V*_T_ / ƒ_p_ | 54.4 (8.9) | 94.2 (19.3) | 63.8 (10.4) | 135.2 (28.3) | 0.69* | 0.14-0.82 | 27.1% | 17.2% | 43.5% |
| MA-1 | *V*_T_ / ƒ_p_ | 87.8 (11.7) | 138.7 (18.7) | 142.1 (23.2) | 202.3 (25.1) | 0.61* | 0.07-0.77 | 55.9% | 62.0% | 45.9% |
| Note: Means across ROIs (±1 Standard Deviation) are depicted for pre- and post-LPS *V*_T_ / ƒ_p_ values. LPS effect % = [(Post-LPS - Pre-LPS) / Pre-LPS] * 100. Partial *η^2^* effect size interpretation: 'Small' ≤ 0.09; 'Moderate' = 0.10-0.24; 'Large' ≥ 0.25. '95% C.I.' refers to the 95% Confidence Interval for the partial *η*^2^ effect size at *p* = .05. Significant LPS effects are noted: **p* ≤ .05. | | | | | | | | | | |

Supplemental Figure Legends

Supplemental Figure 1: Mean AIF data are depicted separately for rs6971 genotype HABs (C/C; gray lines) and MABs (C/T; black lines) pre-LPS (solid lines) and post-LPS (dashed lines).

Supplemental Figure 2: Individual values, pre- and post-LPS, are depicted for each brain region for models that incorporate the AIF: A) 2TCM *V*_T_; B) 2TCM-1k *V*_T_; C) MA-1 *V*_T_ (t*=30); and D) SIME *BP*_P_. The same color marker was used to depict each subject’s data across models and LPS dose (pre- vs. post-LPS).

Supplemental Figure 3: A Time-Activity Curve was extracted from the occipital cortex (OCC) of a representative subject and kinetic model fit are depicted: A) pre-LPS 2TCM; B) post-LPS 2TCM; C) pre-LPS 2TCM-1k; and D) post-LPS 2TCM-1k.
