# Supplementary figures and images for: Quantification of [11C]PBR28 data after systemic lipopolysaccharide challenge
Source: EJNMMI Res. 2020 Mar 12;10:19. doi: 10.1186/s13550-020-0605-7 (PMC7067964; doi:10.1186/s13550-020-0605-7)

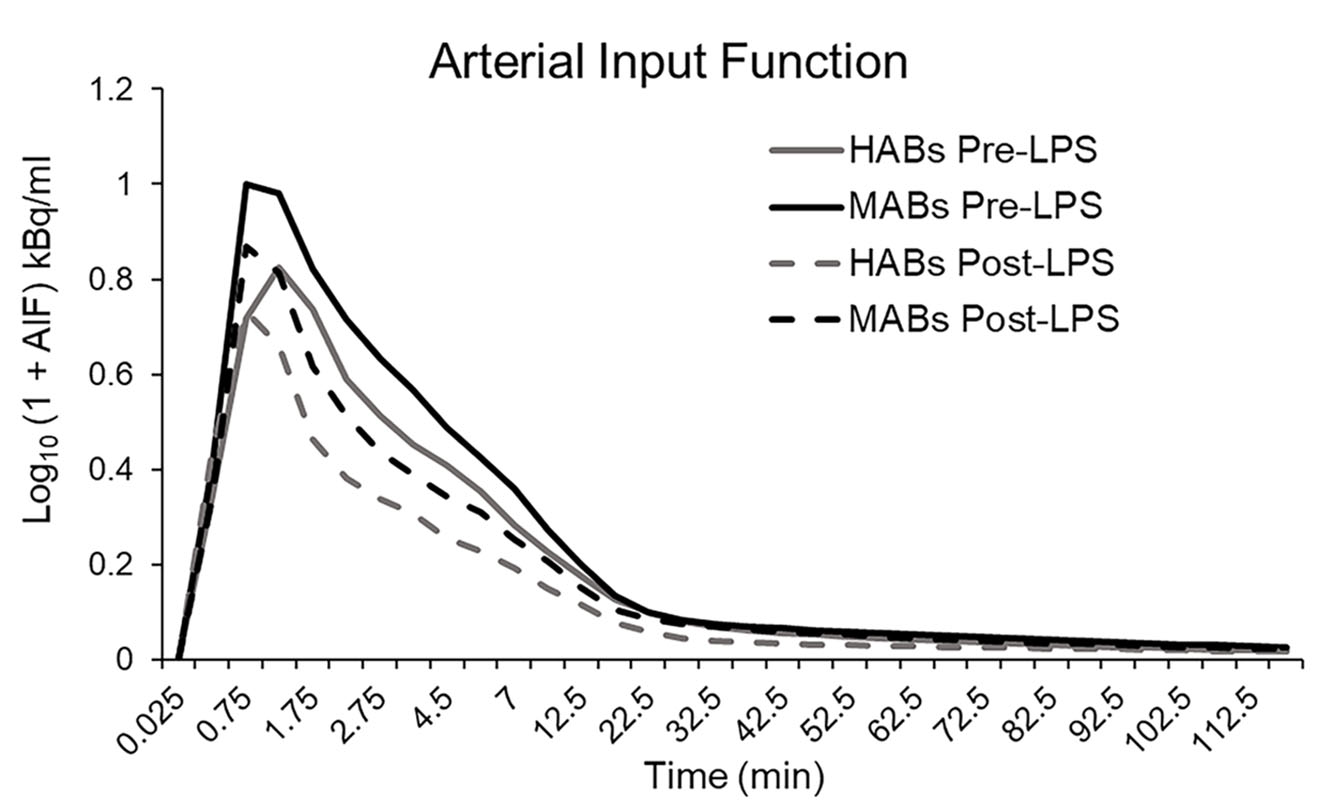

Supplement: Supplementary file 1 — Additional file 1: Supplemental Material. Table S1.Figure S1. Mean AIF data are depicted separately for rs6971 genotype HABs (C/C; gray lines) and MABs (C/T; black lines) pre-LPS (solid lines) and post-LPS (dashed lines). Figure S2. Individual values, pre- and post-LPS, are depicted for each brain region for models that incorporate the AIF: A) 2TCM VT; B) 2TCM-1k VT; C) MA-1 VT (t*=30); and D) SIME BPP. The same color marker was used to depict each subject’s data across models and LPS dose (pre- vs. post-LPS). Figure S3. A Time-Activity Curve was extracted from the occipital cortex (OCC) of a representative subject and kinetic model fit are depicted: A) pre-LPS 2TCM; B) post-LPS 2TCM; C) pre-LPS 2TCM-1k; and D) post-LPS 2TCM-1k. [file 13550_2020_605_MOESM1_ESM.zip › 13550_2020_605_MOESM1_ESM/Supp_Fig_1_new.jpg]

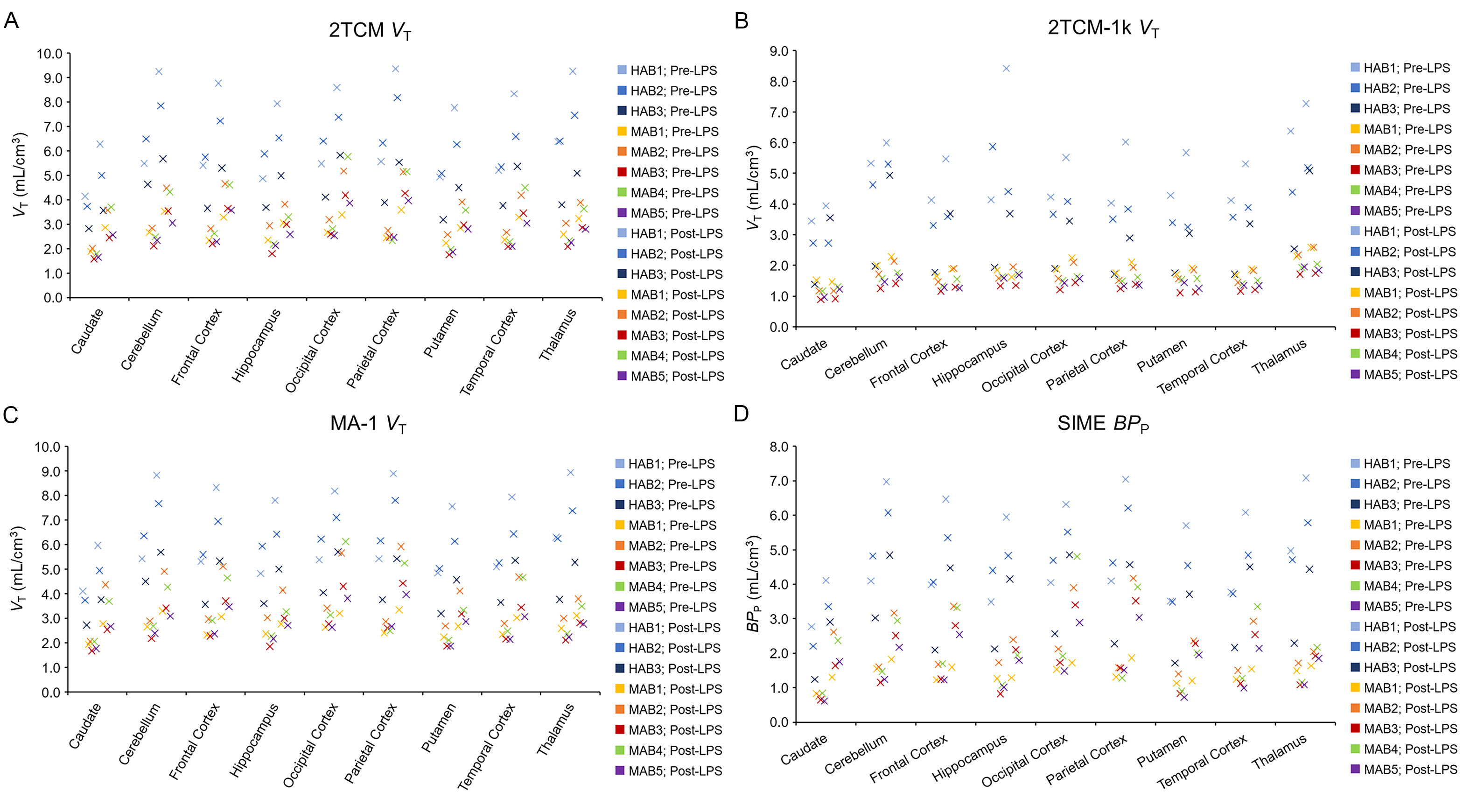

Supplement: Supplementary file 1 — Additional file 1: Supplemental Material. Table S1.Figure S1. Mean AIF data are depicted separately for rs6971 genotype HABs (C/C; gray lines) and MABs (C/T; black lines) pre-LPS (solid lines) and post-LPS (dashed lines). Figure S2. Individual values, pre- and post-LPS, are depicted for each brain region for models that incorporate the AIF: A) 2TCM VT; B) 2TCM-1k VT; C) MA-1 VT (t*=30); and D) SIME BPP. The same color marker was used to depict each subject’s data across models and LPS dose (pre- vs. post-LPS). Figure S3. A Time-Activity Curve was extracted from the occipital cortex (OCC) of a representative subject and kinetic model fit are depicted: A) pre-LPS 2TCM; B) post-LPS 2TCM; C) pre-LPS 2TCM-1k; and D) post-LPS 2TCM-1k. [file 13550_2020_605_MOESM1_ESM.zip › 13550_2020_605_MOESM1_ESM/Supp_Fig_2_new.jpg]

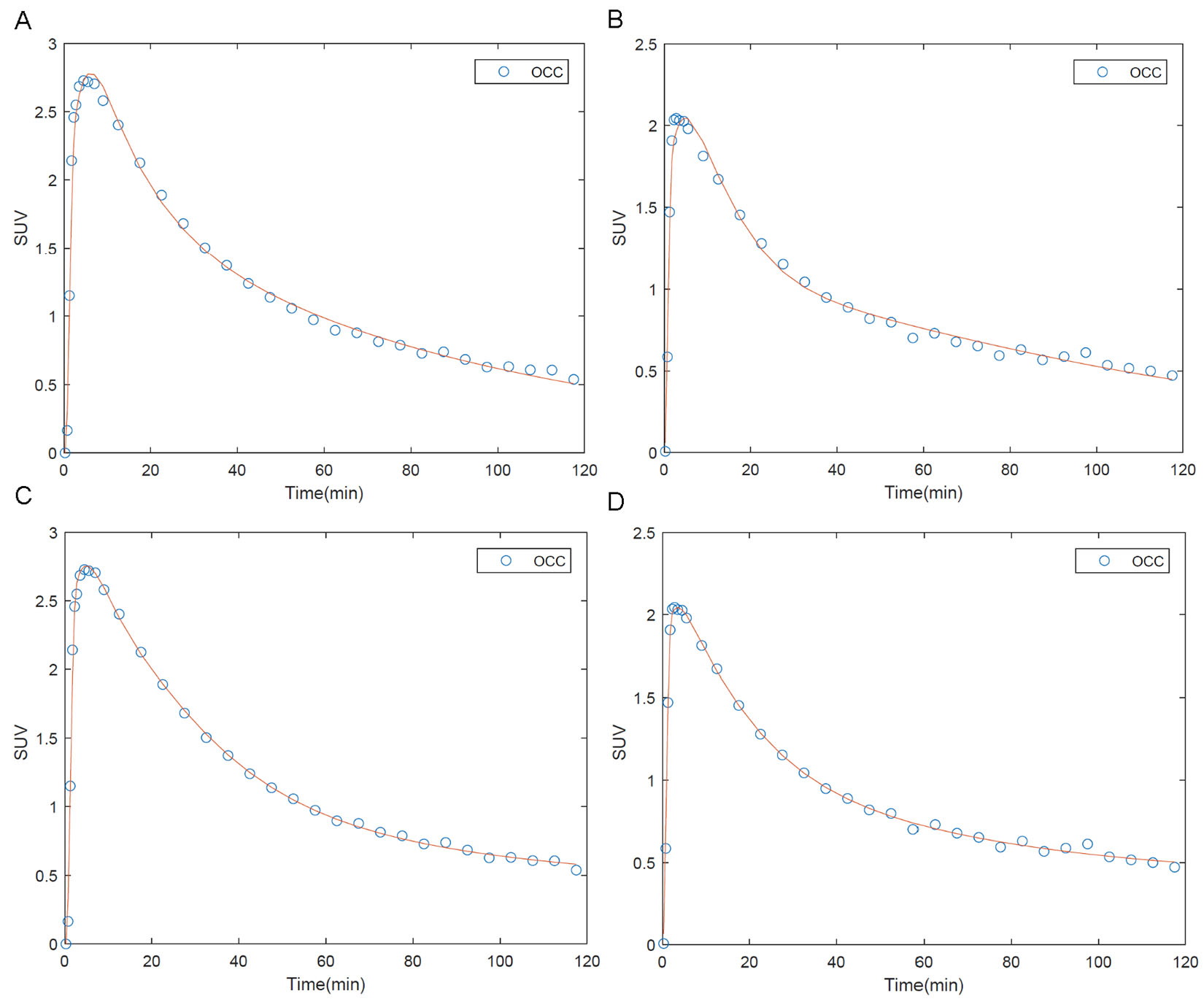

Supplement: Supplementary file 1 — Additional file 1: Supplemental Material. Table S1.Figure S1. Mean AIF data are depicted separately for rs6971 genotype HABs (C/C; gray lines) and MABs (C/T; black lines) pre-LPS (solid lines) and post-LPS (dashed lines). Figure S2. Individual values, pre- and post-LPS, are depicted for each brain region for models that incorporate the AIF: A) 2TCM VT; B) 2TCM-1k VT; C) MA-1 VT (t*=30); and D) SIME BPP. The same color marker was used to depict each subject’s data across models and LPS dose (pre- vs. post-LPS). Figure S3. A Time-Activity Curve was extracted from the occipital cortex (OCC) of a representative subject and kinetic model fit are depicted: A) pre-LPS 2TCM; B) post-LPS 2TCM; C) pre-LPS 2TCM-1k; and D) post-LPS 2TCM-1k. [file 13550_2020_605_MOESM1_ESM.zip › 13550_2020_605_MOESM1_ESM/Supp_Fig_3_new.jpg]
